# PFA fixation enables artifact-free super-resolution imaging of the actin cytoskeleton and associated proteins

**DOI:** 10.1242/bio.019570

**Published:** 2016-07-04

**Authors:** Daniela Leyton-Puig, Katarzyna M. Kedziora, Tadamoto Isogai, Bram van den Broek, Kees Jalink, Metello Innocenti

**Affiliations:** 1 Division of Cell Biology I, The Netherlands Cancer Institute, Plesmanlaan 121, Amsterdam 1066 CX, The Netherlands; 2 Division of Molecular Genetics, The Netherlands Cancer Institute, Plesmanlaan 121, Amsterdam 1066 CX, The Netherlands

**Keywords:** Super-resolution microscopy (SRM), Protein localization, dSTORM, Fixation, Actin cytoskeleton

## Abstract

Super-resolution microscopy (SRM) allows precise localization of proteins in cellular organelles and structures, including the actin cytoskeleton. Yet sample preparation protocols for SRM are rather anecdotal and still being optimized. Thus, SRM-based imaging of the actin cytoskeleton and associated proteins often remains challenging and poorly reproducible. Here, we show that proper paraformaldehyde (PFA)-based sample preparation preserves the architecture of the actin cytoskeleton almost as faithfully as gold-standard glutaraldehyde fixation. We show that this fixation is essential for proper immuno-based localization of actin-binding and actin-regulatory proteins involved in the formation of lamellipodia and ruffles, such as mDia1, WAVE2 and clathrin heavy chain, and provide detailed guidelines for the execution of our method. In summary, proper PFA-based sample preparation increases the multi-color possibilities and the reproducibility of SRM of the actin cytoskeleton and its associated proteins.

## Abstract (precis)

**Summary:** We show that proper PFA fixation allows high-quality super-resolution imaging of the actin cytoskeleton and can outperform gold-standard glutaraldehyde fixation for imaging of actin-binding proteins.

## INTRODUCTION

Single-molecule-based localisation microscopy (SMLM) has circumvented the resolution limit of light microscopy, which prevents resolving details smaller than about half the wavelength of light (Hell, 2009). Consequently, SMLM provides up to 10 times more resolution than classical optical microscopy for biological studies.

Over the last ten years, several SMLM variants have been developed, the most prominent being photoactivated localization microscopy (PALM) (Betzig et al., 2006; Gould et al., 2009), (direct) stochastic optical reconstruction microscopy (STORM and dSTORM) (Rust et al., 2006; Heilemann et al., 2008) and ground-state depletion and single-molecule return (GSDIM) (Folling et al., 2008). Most SMLM methods rely on fluorophores that can be photoactivated or photoswitched by means of light. Depending on the photoactivation or the photoswitching method, SMLM techniques can be divided in ‘targeted switching and readout’ and ‘stochastic switching and readout’ (Hell, 2009; van de Linde et al., 2011).

dSTORM and GSDIM belong to the ‘stochastic switching and readout’ type of SMLM techniques and provide the highest possible resolution among the existing variants. Moreover, these techniques can be enhanced to provide details in the axial direction by inclusion of optical elements that discriminate molecules based on their Z-position (Huang et al., 2008). As dSTORM and GSDIM exploit basic transitions of standard chemical dyes to induce stochastic switching (Folling et al., 2008), they are rarely used in live-cell imaging studies.

SMLM is receiving ever-increasing attention from biologists interested in capturing the distribution of individual molecules within the cell (Endesfelder and Heilemann, 2014; Patterson et al., 2010). Not surprisingly, SMLM is employed to study cellular processes depending on the assembly of monomeric actin into approximately 6 nm-wide filaments, such as formation of membrane protrusions, cell migration, cytokinesis, endocytosis, vesicle trafficking and organelle homeostasis (Isogai et al., 2015; van den Dries et al., 2013; Xu et al., 2012, 2013). Although total internal reflection fluorescence microscopy (TIRFM) allows imaging single actin filaments *in vitro* under conditions that prevent actin-filament bundling, it has insufficient resolution to zoom in to the finest details of F-actin and its interacting proteins. Within cells, diffraction-limited microscopes can neither distinguish single actin filaments from bundles nor precisely map the position of actin-binding proteins along the side and the ends of actin filaments. Electron microscopy (EM) and cryo-electron tomography can readily resolve individual actin filaments in cells, but both methods are very laborious and time-consuming. SMLM bridges the gap between high-throughput, low-resolution conventional microscopy and low-throughput, high-resolution (cryo)EM by visualizing individual actin filaments in cells with intermediate throughput.

However, SMLM is still in its infancy and step-by-step guidelines are only sparsely available (Patterson et al., 2010). In particular, appropriate fixation of the structure of interest remains often very challenging, and more than any other step defines both the quality and the reliability of SMLM images.

The ideal fixative for SMLM should not only preserve the cellular structures faithfully but also allow dense labeling with fast-switching (in)organic fluorophores (Endesfelder and Heilemann, 2014; Ha and Tinnefeld, 2012; van de Linde et al., 2010). For preservation of cellular structure, crosslinking fixatives are usually superior to agents that precipitate and coagulate proteins, such as methanol, ethanol and acids. Crosslinking agents also permit binding of mushroom *Amanita phalloides* toxin Phalloidin to the actin cytoskeleton for very dense labeling of actin (Capani et al., 2001; Wulf et al., 1979).

Two crosslinking agents are commonly used: paraformaldehyde (PFA) and glutaraldehyde (GA). PFA crosslinks amino groups without changing the tertiary structure of proteins so that most epitopes remain available for specific antibodies (Fujiwara, 1980; Robinson and Snyder, 2004). GA cross-links proteins more efficiently than PFA but it has also two main disadvantages: it often makes tertiary structures unrecognizable by antibodies and it penetrates into cells slowly. Thus, cell permeabilization is required either before or during GA fixation, which frequently causes the loss of both cytosolic and cytoskeleton-associated proteins (Robinson and Snyder, 2004).

Although both PFA and GA have been successfully employed to study the actin cytoskeleton through SMLM (van den Dries et al., 2013; Xu et al., 2012; Xu et al., 2013), two recent studies claimed that GA should be the fixative of choice for SMLM of the actin cytoskeleton because PFA did not allow the detection of thin actin bundles and structures (Bachmann et al., 2015; Whelan and Bell, 2015). As the type and concentration of fixative, as well as the incubation time and the permeabilization method, considerably influence the final outcome of the SMLM images, these contrasting results probably reflect the poor standardization of the sample preparation procedures. More importantly, the effects of different fixative agents have not been explored in detail and protocols for the localization of actin-binding and actin-regulatory proteins in SMLM are not available. Thus, anecdotal sample preparation protocols and the lack of a systematic optimization of multi-color SRM seriously limit the flexibility and the reproducibility of SRM.

Here, we show that proper PFA-based sample preparation preserves the architecture of the actin cytoskeleton almost as faithfully as GA and facilitates the localization of various actin-binding and actin-regulatory proteins by SMLM.

## RESULTS

### Proper PFA fixation enables high-quality SMLM imaging of the actin cytoskeleton

**Fig. 1.**
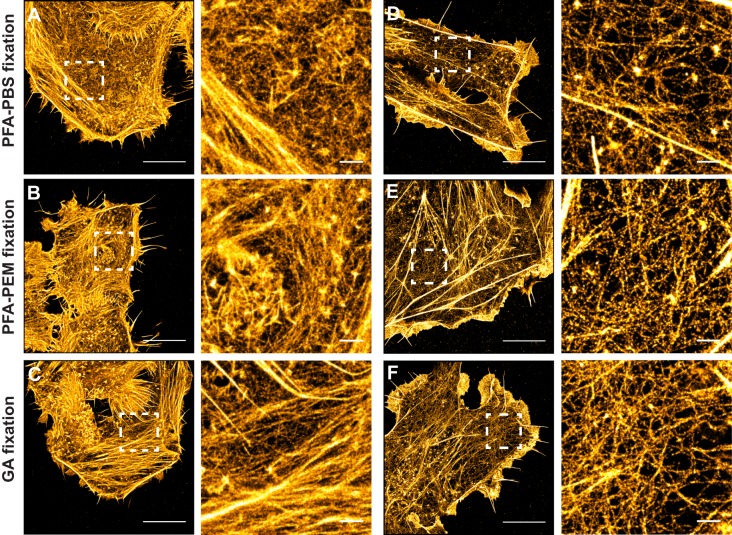
**Proper paraformaldehyde fixation preserves the architecture of the actin cytoskeleton and is compatible with high-quality SMLM.** HeLa (A-C) and Cos-7 (D-F) cells fixed with paraformaldehyde (PFA) dissolved in PBS (A and D), PFA in PEM buffer (B and E) or glutaraldehyde (GA) in cytoskeleton buffer (C and F). All cells were stained with Alexa Fluor-647-labelled Phalloidin and imaged in parallel as described in the Materials and Methods. Representative SMLM images (left) and close ups of the boxed regions (right) are shown. Scale bar A-F: 10 µm, scale bar close ups: 1 µm.

We initially set out to improve a fixation protocol that employs PFA dissolved in PBS (PFA-PBS) and preserves densely packed F-actin bundles but not thin and short actin filaments (Bachmann et al., 2015; Whelan and Bell, 2015). We systematically varied fixation time and temperature, and obtained the best images when the specimen was fixed for 10 min with all washing buffers and PFA kept at 37°C. This allowed visualization of thin actin fibers at SMLM resolution (Fig. 1A,D). Nevertheless, both thin and thick fibers composing the dense cortical actin cytoskeleton of HeLa cells appeared pointillist with this fixation method (Fig. 1A). A slightly less dramatic loss of actin fiber integrity was visible in COS-7 cells, especially in areas with a low-density cortical actin cytoskeleton (Fig. 1D).

We next tested PFA dissolved in PEM buffer, a well-known cytoskeleton-protective buffer (PFA-PEM) (Heuser and Kirschner, 1980). This resulted in a better and more faithful preservation of the actin cytoskeleton with more uniformly stained thin crossing fibers and bundles in both HeLa and COS-7 cells (Fig. 1B,E). Strikingly, these results were comparable to those obtained with GA dissolved in a cytoskeleton-protective buffer (Fig. 1C,F), the gold-standard fixative for high-resolution studies of the actin cytoskeleton (Urban et al., 2010; Xu et al., 2012).

**Fig. 2.**
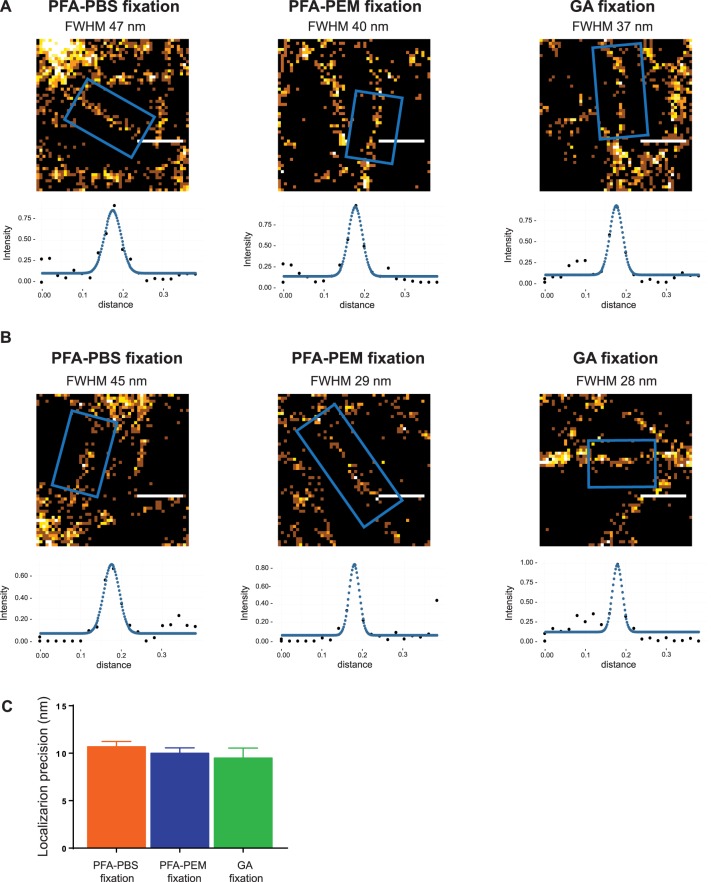
**Proper PFA fixation preserves small actin fibers as faithfully as GA fixation.** (A) PFA-PEM fixation preserves small actin fibers as faithfully as GA fixation. Individual actin fibers (5 to 10 fibers per condition) were manually segmented and their brightness profiles were plotted and fitted to a Gaussian distribution as described in Materials and Methods. Representative fibers, distribution profiles with Gaussian fit and FWHM are shown. Scale bar: 300 nm. (B) Only PFA-PEM and GA fixation preserve very thin actin fibers. Distribution profiles with Gaussian fit and FWHM of the thinnest detected fibers are shown for each fixation method. Scale bar: 300 nm. (C) Localization precision is not affected by the fixation method. Localization precision of images of actin obtained after different fixations was calculated using the Thunderstorm plugin (Ovesny et al., 2014) of Fiji (Schindelin et al., 2012). Bar graph shows localization precision and intensity (mean±s.d.) as obtained from five independent images (PFA-PBS fixation), seven independent images (GA fixation) and nine independent images (PFA-PEM fixation) (one-way ANOVA was employed to compare the obtained results and no significant differences were found).

To compare quantitatively the effects of these fixation protocols on the actin cytoskeleton we measured small actin fibers, as they are most easily lost during fixation. The thinnest detectable fibers were segmented and their brightness profiles fitted to a Gaussian distribution in which full width at half maximum (FWHM) represents the diameter of the fiber. It turned out that the smallest fibers in cells fixed with GA had a diameter of about 35 nm and were thinner than those measured in cells fixed with PFA-PBS (about 45 nm in diameter). Interestingly, the FWHM measured in cells fixed with PFA-PEM was in between the above two values, approximately 40 nm (Fig. 2A). Visual inspection revealed that very thin fibers were only preserved in cells fixed with GA or PFA-PEM (Fig. 2B). Notably, mean uncertainty of the localization of the individual ‘blinks’ of each fluorophore was equivalent for all fixations, showing that imaging conditions (dye, buffer, etc.) were kept constant during all experiments (Fig. 2C). Our results thus show that PFA fixation can yield excellent results to image the actin cytoskeleton at high resolution with SMLM, provided that optimal fixation temperature, time and buffer are employed.

### PFA fixation expands the possibilities of multi-color SMLM of the actin cytoskeleton and actin-binding proteins

We next compared PFA-PEM fixation with GA fixation for preservation of actin-associated proteins and focused on lamellipodia and ruffles, thin veil-like and ephemeral actin-based protrusions that present challenges for SMLM. We analyzed the localization of the actin-regulatory proteins mDia1 and WAVE2, which are involved in the initiation of lamellipodia and ruffles (Isogai et al., 2015).

**Fig. 3.**
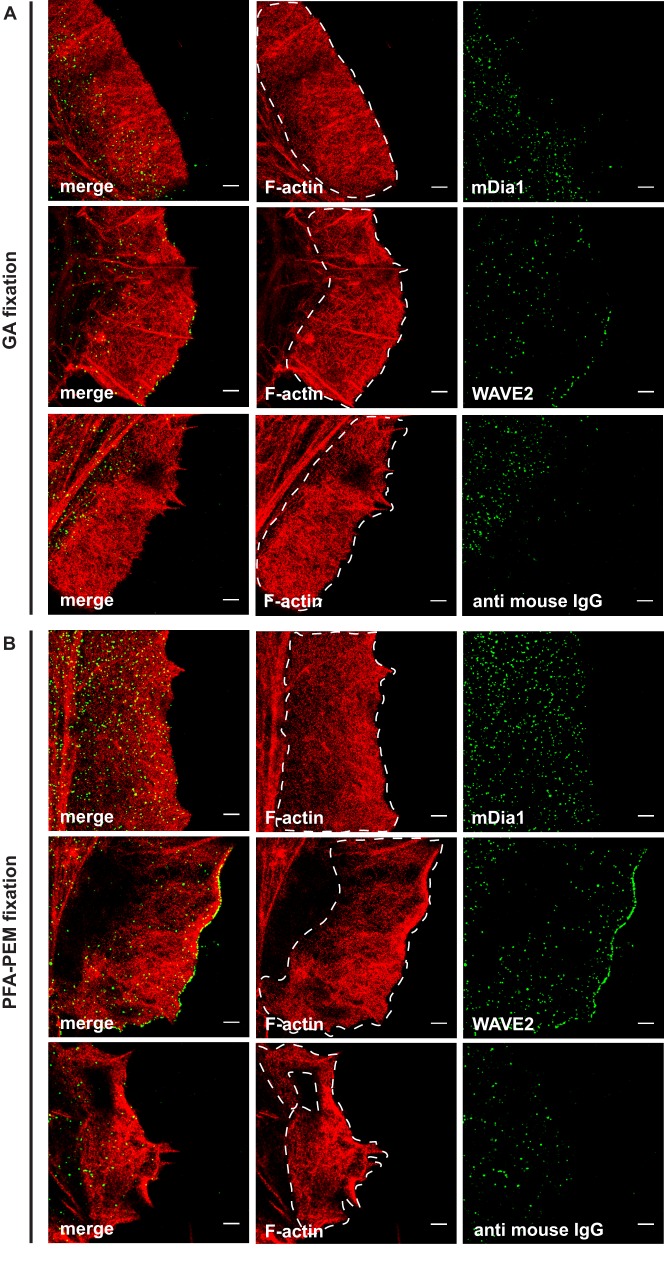
**Paraformaldehyde fixation, but not glutaraldehyde fixation, enables detection of actin-binding proteins within lamellipodia and ruffles.** HeLa cells were stimulated with epidermal growth factor (EGF) (100 ng/ml) for 3 min and fixed with either glutaraldehyde (GA) in cytoskeleton buffer (A) or paraformaldehyde (PFA) in PEM buffer (B) and then stained with either anti-mDia1 (mDia1) or anti-WAVE2 (WAVE2) antibodies or mock stained. All samples were incubated with Alexa Fluor-647-labelled Phalloidin and secondary goat anti-mouse antibodies (anti mouse IgG) labelled with Alexa Fluor-532. Membrane ruffles were imaged in EPI mode. Representative SMLM images show the actin cytoskeleton in red and the actin-binding proteins mDia1 and WAVE2 in green. Dashed white areas depict the region of interest used for the quantification of green particles. Scale bar: 1 µm.

Dual-color SMLM imaging revealed that both GA fixation and PFA-PEM fixation preserved the characteristic actin mesh of lamellipodia and ruffles (Fig. 3A,B). However, density of mDia1 and WAVE2 particles was much higher in the cells fixed with PFA-PEM than in those fixed with GA (Fig. 3A,B). Strikingly, mDia1 signal in GA-fixed cells appeared almost identical to that of cells stained with secondary antibody alone, whereas that of WAVE2 was slightly higher (Fig. 3A). Conversely, PFA-PEM fixation resulted in WAVE2 and mDia1 labeling being much denser than the secondary antibody (Fig. 3B). Importantly, SMLM images from mDia1 knockdown and Nap1 knockdown HeLa cells ruled out that these localizations resulted from the unspecific binding of the primary antibodies (Fig. S1).

These observations were corroborated by quantitative image analysis of the number of particles per area (Table 1). For mDia1, we found that most of the particles imaged after fixation with GA derived from unspecific binding of the secondary antibody, whereas PFA-PEM fixation resulted in an 8-fold increase over the control (Table 1). This and the fact that only few mDia1 particles could be detected in the mDia1 knockdown cells stained with anti-mDia1 antibodies (Fig. S1) jointly indicate that PFA-PEM, but not GA, fixation enables visualizing mDia1 in SMLM. We found no specific mDia1 particles using a second anti-mDia1 antibody that recognizes a different and distant epitope (Fig. S2), thus it appears that mDia1 is washed out on GA fixation. For WAVE2, the number of localized particles per area was higher in the cells fixed with PFA than those fixed with GA (Table 1). Thus, GA fixation did permit specific, yet suboptimal, detection of WAVE2.

**Table 1.**
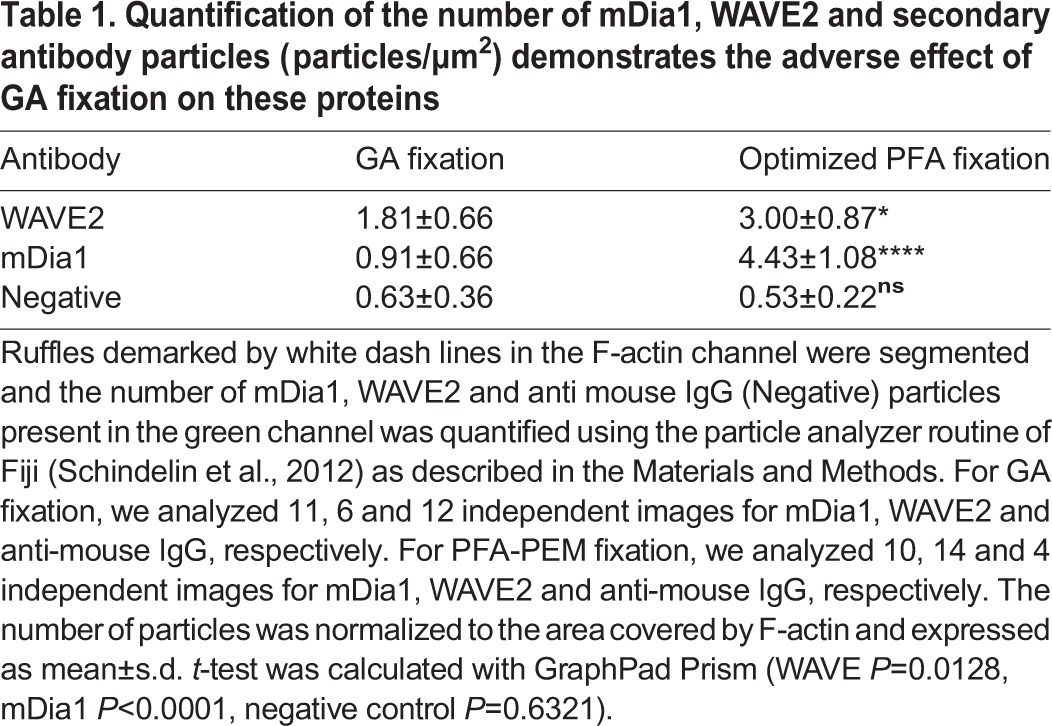
**Quantification of the number of mDia1, WAVE2 and secondary antibody particles (particles/μm^2^) demonstrates the adverse effect of GA fixation on these proteins**

**Fig. 4.**
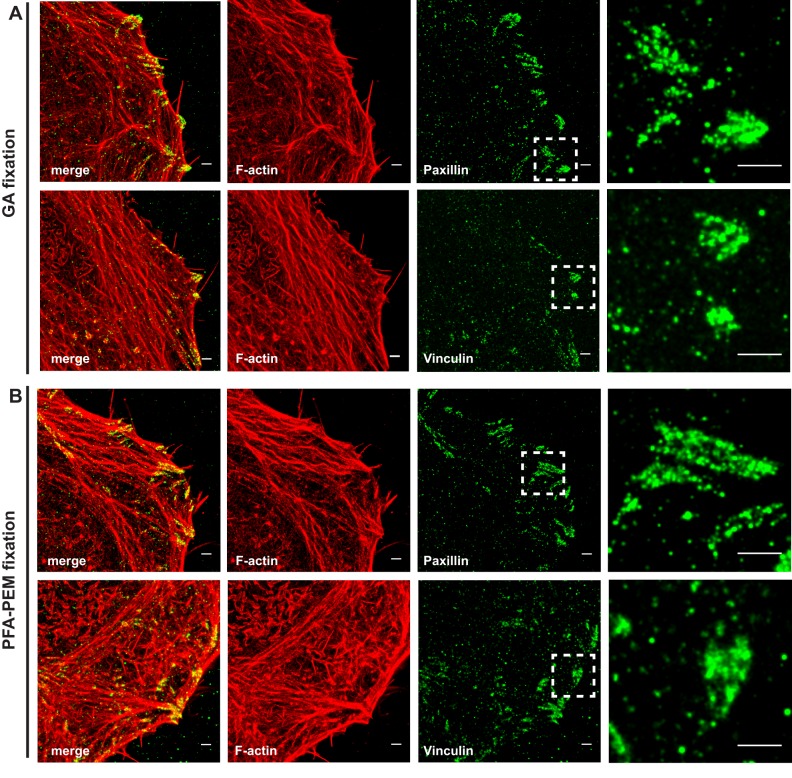
**Paraformaldehyde and glutaraldehyde fixation allow comparable detection of focal adhesion proteins.** HeLa cells were fixed with either glutaraldehyde (GA) in cytoskeleton buffer (A) or paraformaldehyde (PFA) in PEM buffer (B). Cells were stained for Vinculin or Paxillin followed by Alexa Fluor-532-labeled secondary antibodies along with Alexa Fluor-647-labeled Phalloidin. Basal membranes were imaged in TIRF mode as described in the Materials and Methods. Representative SMLM images depict F-actin in red and focal adhesion proteins in green. Dashed white boxes in the Paxillin and the Vinculin images mark the position of the close ups shown on the right. Scale bar: 1 µm.

The different behavior of WAVE2 and mDia1 likely reflects different binding kinetics and/or affinities for F-actin and/or other interacting proteins localizing within lamellipodia and ruffles. Consistent with this notion, focal adhesion-associated proteins Paxillin and Vinculin, which robustly bind to focal adhesion complexes (Geiger et al., 2009), showed a similar localization pattern in both GA-fixed and PFA-PEM-fixed cells (Fig. 4).

These results stress the importance of secondary antibody control experiments to establish labeling specificity. Furthermore, they show that our PFA fixation protocol represents a safe, robust and ready-made alternative for immuno-based multi-color SMLM.

### PFA fixation increases epitope preservation for high-quality SMLM

Clathrin-mediated endocytosis (CME) is a form of actin-based micro-pinocytosis (Galovic et al., 2011; Innocenti et al., 2005; Kaksonen et al., 2006) in which clathrin heavy chain (CHC) assembles around invaginations of the plasma membrane and guides the formation of diffraction-limited vesicles of 90-150 nm (McMahon and Boucrot, 2011). Moreover, CHC has recently been shown to promote the formation of lamellipodia and ruffles through membrane recruitment of WAVE regulatory complex, independently of its role in vesicle trafficking and clathrin light chain (Gautier et al., 2011).

**Fig. 5.**
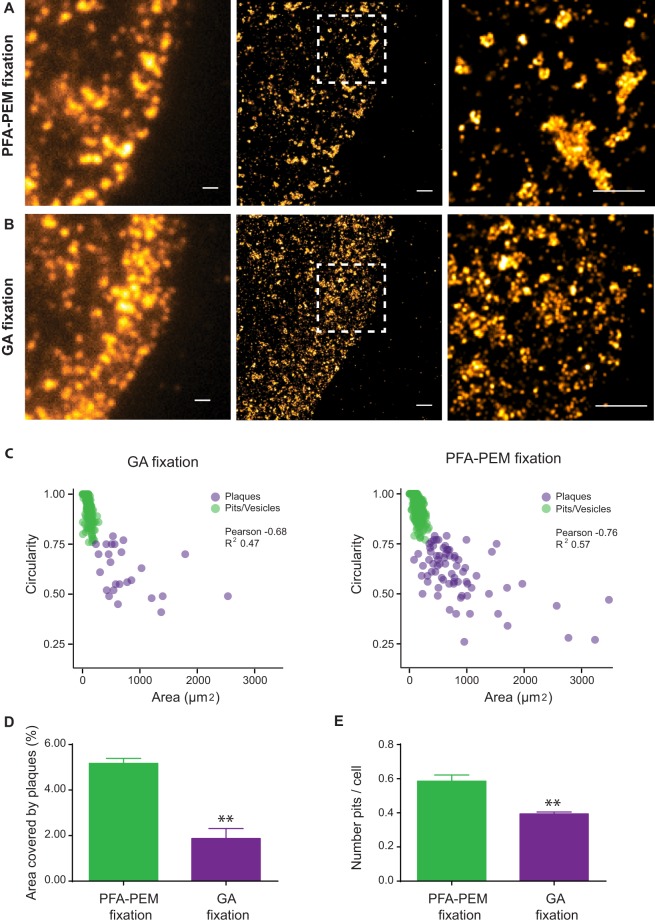
**Paraformaldehyde fixation, but not glutaraldehyde fixation, enables faithful detection of clathrin-coated structures.** HeLa cells were serum starved overnight and then stimulated with EGF (100 ng/ml) for 5 min and fixed with PFA in PEM buffer (A) or glutaraldehyde (GA) in cytoskeleton buffer (B). Cells were stained with anti-clathrin heavy chain (CHC) antibodies and goat Alexa Fluor-647-labeled secondary antibodies. Clathrin-coated structures (CCSs) at the basal plasma membrane were imaged in TIRF mode as described in the Materials and Methods. Representative TIRF images (left), SMLM images (middle) and SMLM close ups (right) corresponding to the dashed white areas are shown. Scale bar: 1 µm. (C) GA fixation perturbs both circular and elongated CCSs. Size of circular and elongated CCSs was obtained by manual segmentation as explained in the Materials and Methods. Scatter plots depict each CCS found in four independent images as a color-coded circle. Note that GA causes a dramatic reduction in the number of CCSs and that the few remaining ones have a smaller size compared to that of the PFA-fixed samples. Pearson coefficients and R^2^ of the correlation between area and circularity were obtained using GraphPad Prism (GA fixation: *P* <0.0001; PFA-PEM fixation: *P* <0.0001). (D,E) Area covered by plaques (D) and number of pits (E) are highly reduced in cells fixed with GA. The area and number of particles are expressed as mean±s.e.m. *t*-test was calculated with GraphPad Prism (D, *P*=0.0025; E, *P*=0.0065, *n*=3).

Using PFA fixation under optimal conditions, we observed two types of clathrin-coated structures (CCSs) in cells by SRM (Fig. 5A): small and round clathrin-coated pits (CCPs) and clathrin-coated vesicles (CCVs) of about 150 nm in size and bigger clathrin patches of more heterogeneous shapes and sizes that likely represent the flat clathrin plaques previously described in HeLa and many other cell lines (Traub, 2009). Conversely, CCPs and CCVs could be hardly located in cells fixed with GA and the few remaining ones appeared very small and non-circular (Fig. 5B). Moreover, the bigger clathrin patches were either absent or lacked clearly defined boundaries (Fig. 5B). Manual and automated morphometric particle analyses of these images showed that GA causes a complete disorganization of the clathrin-coated structures (Fig. 5C and Fig. S3, respectively), with loss of both clathrin coated plaques (Fig. 5D) and pits (Fig. 5E). Surprisingly, only at very high resolution of SMLM were the detrimental effects of GA on CCSs visible as the corresponding TIRFM images appeared very similar (Fig. 5A,B).

As it has been reported that GA destroys the CHC epitope recognized by the anti-CHC X-22 antibody (Collins et al., 2011), the above results suggest that PFA fixation should be preferred to GA fixation for all SMLM applications sensitive to epitope preservation.

## DISCUSSION

During the last few years, several research articles and reviews have stressed that fluorophore switching and localization algorithms are potential sources of SRM image artefacts (Dempsey et al., 2011; Endesfelder and Heilemann, 2014; Hoogendoorn et al., 2014). Even though sample fixation and preparation are as important as the above factors for image quality and resolution, GA-based EM sample preparation protocols are typically employed in SMLM (Xu et al., 2012). As sample preparation protocols for SRM remain anecdotal and have not been optimized, SRM is often challenging and suffers from reproducibility problems.

Here we show that GA-based sample preparation protocols can negatively impact on SMLM imaging and may be severely flawed for immuno-based detection of certain proteins (Fig. 3, Fig. 5). Instead, we present an optimal PFA fixation protocol that provides a simple, flexible and robust way to obtain high-quality super-resolution images of the actin cytoskeleton and associated proteins. This protocol enables proper immuno-based protein localization in two- (or three)-color high-resolution SMLM images and quantitative morphometric and localization analyses.

From the biological point of view, optimal PFA fixation has been instrumental in detecting mDia1 and WAVE2 within lamellipodia and ruffles, thereby providing crucial insight into the mechanism regulating the initiation of these actin-based protrusions. We also anticipate that optimized PFA fixation and SRM will shed new light on the role of CHC in the formation of lamellipodia and ruffles.

In summary, washout and epitope masking of actin-binding and actin-associated proteins during GA fixation are unrecognized fundamental technical pitfalls of SMLM. As optimized PFA-based sample preparation represents a substantial technical advance that increases the possibilities and the reproducibility of multi-color SRM, our protocol and an accompanying troubleshooting table will be deposited in a freely accessible public repository (Protocol Exchange).

## MATERIALS AND METHODS

### Reagents

Albumin Bovine Fraction V, pH 7.0, was from Serva (Huissen, The Netherlands), 2-butanol, glutardialdehyde solution 25%, magnesium chloride hexahydrate, paraformaldehyde (PFA), sodium hydroxide pellets, Triton X-100 were from Merck (Amsterdam, The Netherlands), calcium chloride dehydrate, catalase from *Aspergillus niger*, Dulbecco's modified Eagle's medium, EGTA, gelatin from porcine skin (300 g Bloom), glucose oxidase ethanol, hydrochloric acid, MES hydrate, phosphate buffered saline (PBS) tablets, petroleum ether, sodium borohydride were from Sigma-Aldrich (Zwijndrecht, The Netherlands). Cysteamine hydrochloride–MEA, D-(+)-glucose anhydrous were from Fluka (Zwijndrecht, The Netherlands). Murine epidermal growth factor (EGF) was from Invitrogen (Breda, The Netherlands). Fetal bovine serum (FBS) was from APS (Bedford, UK). PIPES was from Fisher Scientific (Breda, The Netherlands). Precision tissue wipes were from Kimtech Science (Ede, The Netherlands).

### Antibodies

Phalloidin-Alexa Fluor 647 was from Invitrogen (Zwijndrecht, The Netherlands), mouse anti-Clathrin heavy chain antibody (X22) was from Thermo Scientific (Breda, The Netherlands), mouse anti-mDia1 and mouse anti-paxillin were from BD Transduction Laboratories (Breda, The Netherlands), mouse anti-mDia1 (D3) was from Santa Cruz (Heidelberg, Germany), mouse anti-tubulin was from Sigma (Zwijndrecht, The Netherlands), rabbit anti-vimentin was from GeneTex (Irvine, CA, USA), mouse anti-WAVE2 antibody was generated in-house (Isogai et al., 2015).

### Inmunofluorescence

Previous to all steps reagents were filter sterilized. 24 mm #1.5 coverslips were coated with 0.5% gelatin solution in a humidified incubator at 37°C for 30 min. Cells were plated on gelatin-coated coverslips and grown in DMEM supplemented with 10% FCS. The next day they were either serum starved overnight in DMEM supplemented with 0.1% FCS or kept left untreated. For Fig. 2, cells were stimulated for 3 min with 100 ng/ml EGF. For Fig. 3, cells were stimulated for 5 min with 100 ng/ml EGF.

For GA fixation cells were briefly rinsed with pre-warmed PBS supplemented with magnesium and calcium and incubated for 2 min in 0.3% GA and 0.25% Triton X-100 in cytoskeleton-preserving buffer (10 mM MES pH 6.1, 150 mM NaCl, 5 mM EGTA, 5 mM glucose, 5 mM MgCl_2_) followed by 8 min in 0.5% GA in cytoskeleton-preserving buffer. Cells were then rinsed with and incubated for 7 min in freshly prepared 0.1% NaBH_4_ in PBS (Xu et al., 2012).

For PFA fixation, a 20% (w/v) stock solution was prepared from powder as follows: PFA was dissolved in warm ddH_2_O under constant stirring keeping the temperature below 55-57°C. NaOH (10 N) drops were added to help PFA dissolve. pH was adjusted to 7.2 by adding HCl (37%). The stock solution was filter-sterilized and kept at −20°C for up to a year (Hrckova and Olson, 2013). Cells were rinsed briefly with pre-warmed PBS containing calcium and magnesium and incubated for 10 min in freshly prepared pre-warmed 4% solution of PFA dissolved in PBS or cytoskeleton-preserving buffer (PEM) (80 mM PIPES pH 6.8, 5 mM EGTA, 2 mM MgCl_2_) (optimal PFA fixation). Cells were rinsed twice with PBS and incubated for 10 min in 0.5% Triton X-100 in PBS.

In all cases, cells where incubated in 5% BSA for at least one hour at 37°C or overnight at 4°C. Primary antibodies were diluted in 5% BSA and samples were incubated for 45 min at room temperature. Secondary antibodies and Phalloidin were incubated for 30 min. Phalloidin was used coupled with Alexa Fluor-647 at a final concentration of 0.6 U of Phalloidin (Molecular Probes for Life Technologies). Goat anti-mouse and goat anti-rabbit secondary antibodies coupled Alexa Fluor-532 (Molecular Probes for Life Technologies, Breda, The Netherlands) were used a final concentration of 10 µg/ml.

### Super resolution imaging and localization analysis

Specimens were imaged with a Leica SR-GSD 3D microscope using an oxygen scavenging system (GLOX: 10% glucose+0.5 mg ml^−1^ glucose oxidase+40 µg ml^−1^ catalase) supplemented with a reducing agent (cysteamine hydrochloride-MEA) at 100 mM. Images were taken in TIRF or EPI mode at 10 ms or 28 ms exposure time for 10,000-15,000 frames. Structured background subtraction with a temporal median filter (Hoogendoorn et al., 2014) was performed on the blinking movies using home-built software and the resulting movies were analyzed with Thunderstorm (Ovesny et al., 2014), an open source plugin for ImageJ (Schneider et al., 2012) (ImageJ; http://imagej.nih.gov/ij/), to locate the center of blinking molecules, correct the XY drift and construct the final image. Images were rendered with 20 nm pixel size, with the Normalized Gaussian visualization option. For figure visualization purposes, images were convolved with a mean filter of 3×3 pixels. Images of filaments used for brightness profile plotting (Fig. 2) were rendered with 20 nm pixel size with the Histogram visualization option. Chromatic aberration (CA) was corrected using images obtained from 0.1 μm diameter Tetraspec microspheres (Invitrogen) embedded in a matrix. An affine transformation matrix was constructed from those data and affine correction of all images was carried out with an ImageJ macro, using the plugin Image Stabilizer (http://www.cs.cmu.edu/~kangli/code/Image_Stabilizer.html).

### Quantification of the FWHM of small actin fibers

Images were rendered with 20 nm pixel size, with the Histogram visualization option. Small actin fibers were selected manually. Brightness profiles were acquired with an imageJ macro as follows: lines with thickness 20 pixels (straight or curved) were drawn manually on the fibers and the underlying pixels were excised from the images. Obtained Images from curved lines were straightened. Profiles of these straight filaments were fit with a 1D Gaussian function with offset. The FWHM was defined as 2.35 times the sigma (width) of the Gaussian fit.

### Quantification of the distribution of particles of WAVE2, mDia1 and anti-mouse IgG

Ruffles were segmented using the Phalloidin images. WAVE2-, mDia1- or secondary antibody-positive particles were quantified with ImageJ (Schneider et al., 2012) (ImageJ; http://imagej.nih.gov/ij/) using the analyze particles routine and selecting the particles bigger than 2 pixels with at least two localizations per pixel. The relative abundance of these particles was calculated by dividing the obtained number by the total area of the ruffle (particles/μm^2^).

### Quantification of the distribution of pits and plaques

Morphometric analysis of clathrin-coated structures was performed by manual image segmentation. Selected particles were characterized with ImageJ (Schneider et al., 2012) (ImageJ; http://imagej.nih.gov/ij/) using the analyze particles routine. For the automated analysis of all particles, particles bigger than 4 pixels were characterized for area and circularity with ImageJ (Schneider et al., 2012) (ImageJ; http://imagej.nih.gov/ij/) using the analyze particles routine.

### Generation of stable knockdown cells

Stable knockdown cells were described and published before (Beli et al., 2008; Isogai et al., 2015).

### Statistical analyses

GraphPad Prism (version 6.oh) was used to carry out all statistical analyses.
